# Mesenchymal stem cells induce endothelial cell quiescence and promote capillary formation

**DOI:** 10.1186/scrt412

**Published:** 2014-02-17

**Authors:** Torbjorn O Pedersen, Anna L Blois, Ying Xue, Zhe Xing, Yang Sun, Anna Finne-Wistrand, James B Lorens, Inge Fristad, Knut N Leknes, Kamal Mustafa

**Affiliations:** 1 Department of Clinical Dentistry, Center for Clinical Dental Research, University of Bergen, Årstadveien 19, N-5009 Bergen, Norway; 2 Department of Biomedicine, University of Bergen, Bergen, Norway; 3 Department of Clinical Medicine, Centre for Cancer Biomarkers, Section for Pathology, University of Bergen, Bergen, Norway; 4 Vascular Biology Department, Children’s Hospital Boston, Harvard Medical School, Boston, USA; 5 Department of Fibre and Polymer Technology, KTH Royal Institute of Technology, Stockholm, Sweden

## Abstract

**Introduction:**

Rapid establishment of functional blood vessels is a prerequisite for successful tissue engineering. During vascular development, endothelial cells (ECs) and perivascular cells assemble into a complex regulating proliferation of ECs, vessel diameter and production of extracellular matrix proteins. The aim of this study was to evaluate the ability of mesenchymal stem cells (MSCs) to establish an endothelial-perivascular complex in tissue-engineered constructs comprising ECs and MSCs.

**Methods:**

Primary human ECs and MSCs were seeded onto poly(L-lactide-co-1,5-dioxepan-2-one) (poly(LLA-co-DXO)) scaffolds and grown in dynamic culture before subcutaneous implantation in immunocompromised mice for 1 and 3 weeks. Cellular activity, angiogenic stimulation and vascular assembly in cell/scaffold constructs seeded with ECs or ECs/MSCs in a 5:1 ratio was monitored with real-time RT-PCR, ELISA and immunohistochemical microscopy analysis.

**Results:**

A quiescent phenotype of ECs was generated, by adding MSCs to the culture system. Decreased proliferation of ECs, in addition to up-regulation of selected markers for vascular maturation was demonstrated. Baseline expression of VEGFa was higher for MSCs compared with EC (*P* <0.001), with subsequent up-regulated VEGFa-expression for EC/MSC constructs before (*P* <0.05) and after implantation (*P* <0.01). Furthermore, an inflammatory response with CD11b + cells was generated from implantation of human cells. At the end of the 3 week experimental period, a higher vascular density was shown for both cellular constructs compared with empty control scaffolds (*P* <0.01), with the highest density of capillaries being generated in constructs comprising both ECs and MSCs.

**Conclusions:**

Induction of a quiescent phenotype of ECs associated with vascular maturation can be achieved by co-seeding with MSCs. Hence, MSCs can be appropriate perivascular cells for tissue-engineered constructs.

## Introduction

In tissue engineering, inadequate vascular supply is a limiting factor for the reconstruction of larger defects. The incorporation of bioengineered microvessels derived from vascular endothelial cells (ECs) in cell/scaffold constructs, is an approach attempting to ensure appropriate oxygenation of developing tissue. However, bioengineered vasculature must reach a physiologically functional level in order to supply oxygen and nutrients effectively and depends on supporting cells for stability and maintenance [1].

Mesenchymal stem cells (MSCs) are investigated primarily as progenitor cells capable of regenerating bone, cartilage and adipose tissue, thus being an attractive cell source for tissue engineering. However, implantation of cells into a hypoxic microenvironment might hamper the success of cell based tissue regeneration, and increasing efforts to pre-vascularize constructs with ECs have, therefore, been made.

Milestone discoveries in the study of MSCs are the fibroblastic colony-forming units initiated by single cells [2], the multilineage potential [3] and, more recently, a shift in view towards MSCs as tissue-specific progenitors residing in close proximity to the microvasculature of various organs [4]. The latter might serve as a rationale for MSCs as a potential perivascular cell in pre-vascularized constructs for tissue regeneration.

Pericytes are vascular mural cells of microvessels residing alongside ECs within the vascular basement membrane [5]. In the process of blood vessel development, perivascular cells are recruited to tubular ECs, and the endothelial-perivascular complex produces extracellular basement membrane proteins leading to increasingly mature blood vessels [6]. The transition from vascular morphogenesis to a stabilized vasculature, therefore, depends on perivascular cells [7], the absence of which might lead to non-functional immature vessels [8]. Perivascular cells establish specific focal contacts with the adjacent endothelium [5], and it has been shown that their presence is beneficial for EC survival [9]. In fact, interactions with pericytes might regulate EC proliferation and differentiation [5]. Mesenchymal precursor cells have shown the ability to differentiate into smooth muscle cells (SMCs) [10,11], but the ability of MSCs to differentiate into non-skeletal lineages is controversial [4]. Although SMCs can support the development of functional engineered microvessels [12], they lack the ability to regenerate skeletal tissues. MSCs might, therefore, be a more appropriate cell for tissue engineering applications, where a functional vasculature alongside cells with regenerative potential is required. Previous studies have shown that endothelial microvascular networks can be generated *in vitro* with supportive cells other than SMCs, including fibroblasts and MSCs [13-15].

This study assessed the effect of MSCs on the vascular endothelium in two- and three-dimensional culture systems, as well as after subcutaneous implantation. Results show that quiescent ECs and vascular maturation was induced by the presence of MSCs. Furthermore, both cellular constructs induced higher vascular density than control scaffolds, with the highest density of capillaries generated from co-implantation of ECs and MSCs.

## Methods

### Cell expansion and characterization

Human umbilical vein endothelial cells were purchased from Lonza (Clonetics®, Walkersville, MD, US) and expanded in endothelial cell growth medium (EGM®) (Lonza) containing 500 ml endothelial cell basal medium and supplements: Fetal bovine serum (FBS) 10 ml, bovine brain extract (BBE) 2 ml, human epidermal growth factor (hEGF) 0.5 ml, hydrocortisone 0.5 ml and GA-1000 0.5 ml. Primary human bone-marrow derived stem cells (MSCs) were purchased from StemCell Technologies (Vancouver, BC, Canada), and expanded in MesenCult® complete medium (StemCell Technologies). The purity of MSCs was evaluated by flow cytometry, were it was found that >90% of the cells expressed CD29, CD44, CD105 and CD166 while <1% expressed CD14, CD34 and CD45. Cells no older than passage five were used, and all cells were cultured at 37°C and 5% CO_2_.

### Two- and three-dimensional culture systems

To investigate morphology *in vitro*, 10^5^ ECs were cultured in six-well plates (NUNC, Roskilde, Denmark) in mono-culture or in co-culture with MSCs in a 5:1 ratio in EGM® culture medium (Lonza). After one week, ECs were stained with TRITC-conjugated UEA-1 (Sigma-Aldrich, St. Louis, MO, US) and MSCs with FITC mouse anti-human CD90 (BD Biosciences, San Jose, CA, US). In order to determine the number of viable ECs, cells in co-culture were separated with CD31 Endothelial Cell Dynabeads® (Invitrogen, Carlsbad, CA, US) according to the manufacturer’s instructions. ECs were stained with 0.4% trypan blue and counted with a Countess® Automated Cell Counter (Invitrogen).

Previous publications have described the production of poly(LLA-*co*-DXO) scaffolds [16], and the seeding of cell/scaffold constructs for *in vivo* implantation [17]. Briefly, scaffolds were incubated overnight in EGM® (Lonza) culture medium. The next day, each scaffold was seeded with 5 × 10^5^ cells, ECs or ECs/MSCs in a 5:1 ratio. An orbital shaker (Eppendorf®, Hamburg, Germany) was applied for five minutes at 1,000 rpm to facilitate homogenous distribution of cells [18]. Scaffolds were transferred to separate spinner flask bioreactors (Wheaton Science, Millville, NJ, US) the next day and grown in dynamic three-dimensional culture at 50 rpm for one week in EGM® culture medium (Lonza) for both experimental groups. A dermal skin puncher was used to puncture six mm discs, which were either implanted *in vivo* or processed for analysis.

### Surgical implantation

All animal experiments were approved by the Norwegian Animal Research Authority (Local approval number: 3029) and conducted according to the European Convention for the Protection of Vertebrates used for Scientific Purposes. Sixteen non-obese severe combined immunodeficient (NOD/SCID) mice (Gade Institute, University of Bergen/Taconic Farms) six- to eight-weeks old were used for implantation of cell/scaffold constructs. An intramuscular injection of 20 μl 1:2 Rompun (Xylazin, 20 mg/ml) (Bayer Health Care, Leverkusen, Germany) and Narketan (Ketamin) (Vétoquinol, Lure, France) was given to anesthetize the animals. A 2.5 cm incision was made on the back of each animal, providing sufficient space for subcutaneous implantation of scaffolds. Vetbond™ Tissue Adhesive (n-butyl cyanoacrylate) (3M™, St. Paul, MN, US) was applied for wound closure. Samples were retrieved after one and three weeks implantation, following euthanasia with deep isoflurane anesthesia (Schering Plough, Kenilworth, NJ, US) and cervical dislocation. Depending on the following analysis, samples were either frozen in liquid nitrogen (RNA-isolation), fixed in 4% paraformaldehyde (PFA) (paraffin embedding) or embedded in optimal cutting temperature (OCT) tissue-tech (Sakura Finetek, Tokyo, Japan) (cryosectioning).

### Real-time RT-PCR

For *in vitro* and *in vivo* evaluations of gene expression, extraction of RNA was performed with an E.Z.N.A.® Total RNA Kit (Omega Bio-Tek, Norcross, GA, US). A Nanodrop Spectrophotometer (ThermoScientific NanoDrop Technologies, Wilmington, DE, US) was used to quantify and evaluate RNA purity. A high capacity cDNA Archive Kit (Applied Biosystems, Carlsbad, CA, US) was applied for reverse transcription reactions, where total RNA (1,000 ng) was mixed with nuclease free water, reverse transcriptase buffer, random primers, dNTP and MultiScribe reverse transcriptase according to the manufacturer’s instructions. Real-time RT-PCR was performed on a StepOne™ real time PCR system (Applied Biosystems) with standard enzyme and cycling conditions, and cDNA corresponding to 10 ng mRNA in each reaction, prepared in duplicate for each target gene. Human Taqman® gene expression assays were angiopoietin-1 (ANG-1), angiopoietin-2 (ANG-2), α-smooth muscle actin (α-SMA), SM22α, ki67, proliferating cell nuclear antigen (PCNA), CD31, von Willebrand Factor (vWF), vascular endothelial growth factor-a (VEGFa), fibroblast growth factor-1 (FGF-1), platelet derived growth factor-b (PDGFb) and macrophage colony stimulating factor-1 (CSF-1). Mouse Taqman® gene expression assays were VEGFa, ANG-1, ANG-2, α-SMA and ankyrin repeat domain 1 (ANKRD1).

### Histological evaluation

Embedding with OCT tissue-tech (Sakura Finetek) was done before samples were frozen with 2-methylbutan ReagentPlus® (Sigma-Aldrich, St. Louis, MO, US) and liquid nitrogen. A Leica CM 3050S microtome (Leica Microsystems, Wetzlar, Germany) was used to make 8 μm sections at -24°C. Fixation in 4% PFA was used for samples intended for paraffin sectioning before embedding. Sections acquired from the middle part of the samples were deparaffinized and stained with hematoxylin and eosin. CD11b- and CD31-staining was performed with rat anti-mouse primary antibodies (BD Biosciences, San Jose, CA, US) in a 1:200 dilution for four hours at room temperature, with TRITC-conjugated goat anti-rat secondary antibody in a 1:500 dilution for two hours. Circumference and area fraction of individual vessels were outlined using NIS-Elements, BR 3.07 software (Nikon, Tokyo, Japan). A Nikon 80i microscope (Nikon) was used for imaging.

### ELISA

A commercially available human VEGFa enzyme-linked immunosorbent assay (R&D Systems, Minneapolis, MN, US) was applied for protein analysis of *in vitro* samples according to the manufacturer’s instructions. Absorbance was measured with a FLUOstar OPTIMA microplate reader (BMG LABTECH, Ortenberg, Germany) and the values were compared to a known standard curve.

### Statistical analysis

For all statistical tests, *P* <0.05 was defined as a significant difference. The independent samples t-test was applied for two-group comparisons, whereas a multiple comparison one-way analysis of variance (ANOVA) was applied to output parameters that included three experimental groups. The Brown-Forsythe test was performed for equality of variances with no significant differences between the groups. To quantify vessels, five images were obtained systematically at 20x magnification for each scaffold (N = 3 for each group). For real time RT-PCR data a comparative Ct method with GAPDH as endogenous control was used, with statistical differences calculated between mean RQ-values for each experimental group. Data are presented as mean ± standard deviation (N = 3). Statistical analysis was performed with SPSS Statistics 19.0 (IBM, Armonk, NY, US) and GraphPad Prism Version 6.0 (GraphPad, San Diego, CA, US).

## Results

### Mesenchymal stem cells induce endothelial cell quiescence

**Figure 1:**
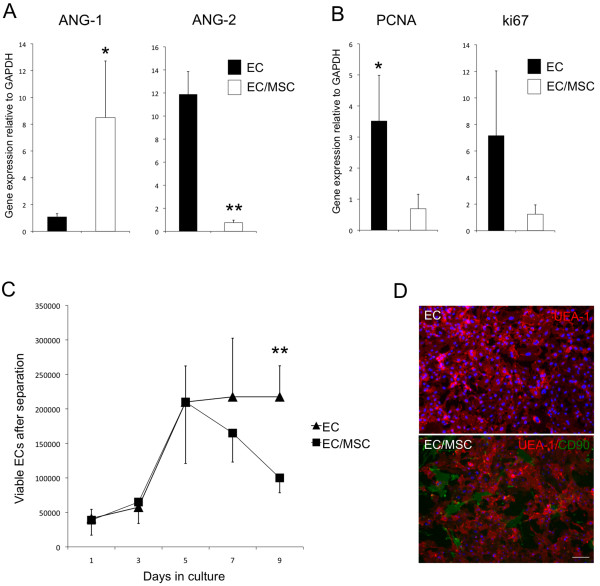
**Mesenchymal stem cells induce endothelial cell quiescence. (A)** The relative gene expression of ANG-1 and ANG-2 from mono- and co-cultured constructs after one week dynamic culture *in vitro* was altered by co-seeding endothelial cells (ECs) with mesenchymal stem cells (MSC). An up-regulation of ANG-1 and a down-regulation of ANG-2 was detected. **(B)** Also, cell proliferation markers PCNA and ki67 were both down-regulated in EC/MSC-constructs. **(C)** In a two-dimensional co-culture system, which allowed separation of cells, the number of viable ECs was reduced in the presence of MSCs after five days culture. After seven days the difference was not statistically significant (*P* = 0.07), whereas after nine days the number of viable ECs was significantly reduced in the presence of MSCs. **(D)** UEA-1 staining (red) demonstrated a quiescent phenotype of ECs in co-culture with CD90-positive MSCs (green) (10x). Nuclei were stained with DAPI. Scale bar = 20 μm. * = *P* <0.05, ** = *P* <0.01. ANG-1, angiopoietin-1; ANG-2, angiopoietin-2; DAPI, 4',6-diamidino-2-phenylindole; PCNA, proliferating cell nuclear antigen.

The expression of endothelial specific ANGs was altered by the addition of MSCs to the culture system (Figure 1A). An up-regulation of ANG-1 was observed in co-cultured cells when compared with controls of mono-cultured ECs. The opposite regulation of ANG-2 was found, with a 12-fold decrease in expression for constructs comprising both ECs and MSCs (*P* <0.01). Cell proliferation was assessed through expression of PCNA and ki67, both being down-regulated in EC/MSC constructs relative to the mono-cultured ECs (Figure 1B).

In a two-dimensional culture system where cells could be separated, a cell count was performed to assess the number of viable ECs in the two experimental groups over a period of nine days (Figure 1C). A reduced number of ECs was found after seven days, but this was not statistically significant (*P* = 0.07). After nine days co-culture the number of viable ECs was significantly reduced in the presence of MSCs. Mono-cultured ECs showed a typical cobblestone-like morphology after one week of two-dimensional culture *in vitro*, and with the addition of MSCs a less proliferative phenotype could be observed (Figure 1D). ECs did not organize into microvascular networks in the presence of MSCs at this ratio (5:1 EC/MSC).

### Expression of vascular and perivascular biomarkers before and after implantation

**Figure 2:**
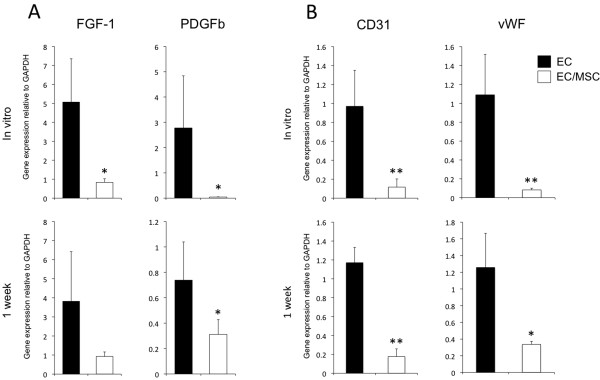
**Human specific expression of vascular biomarkers before and after implantation. (A)** The relative gene expression of the human specific vascular biomarkers fibroblast growth factor-1 (FGF-1) and platelet derived growth factor-b (PDGFb) was down-regulated in co-culture at implantation after one week of dynamic three-dimensional culture *in vitro* and one week after implantation *in vivo*. **(B)** The expression of human specific endothelial cell markers CD31 and von Willebrand Factor (vWF) was also down-regulated in co-culture both before and one week post-implantation. * = *P* <0.05, ** = *P* <0.01.

FGF-1 and PDGFb are factors involved in angiogenic sprouting and the recruitment of pericytes to developing blood vessels. Both showed higher expression in the mono-culture group at implantation and one week post-implantation (Figure 2A). In order to determine the influence of MSCs on EC quiescence, the expression of CD31 and vWF was analyzed (Figure 2B). In EC/MSC constructs, a down-regulation of both markers was found after one week of culture *in vitro*, as well as one week after *in vivo* implantation.

**Figure 3:**
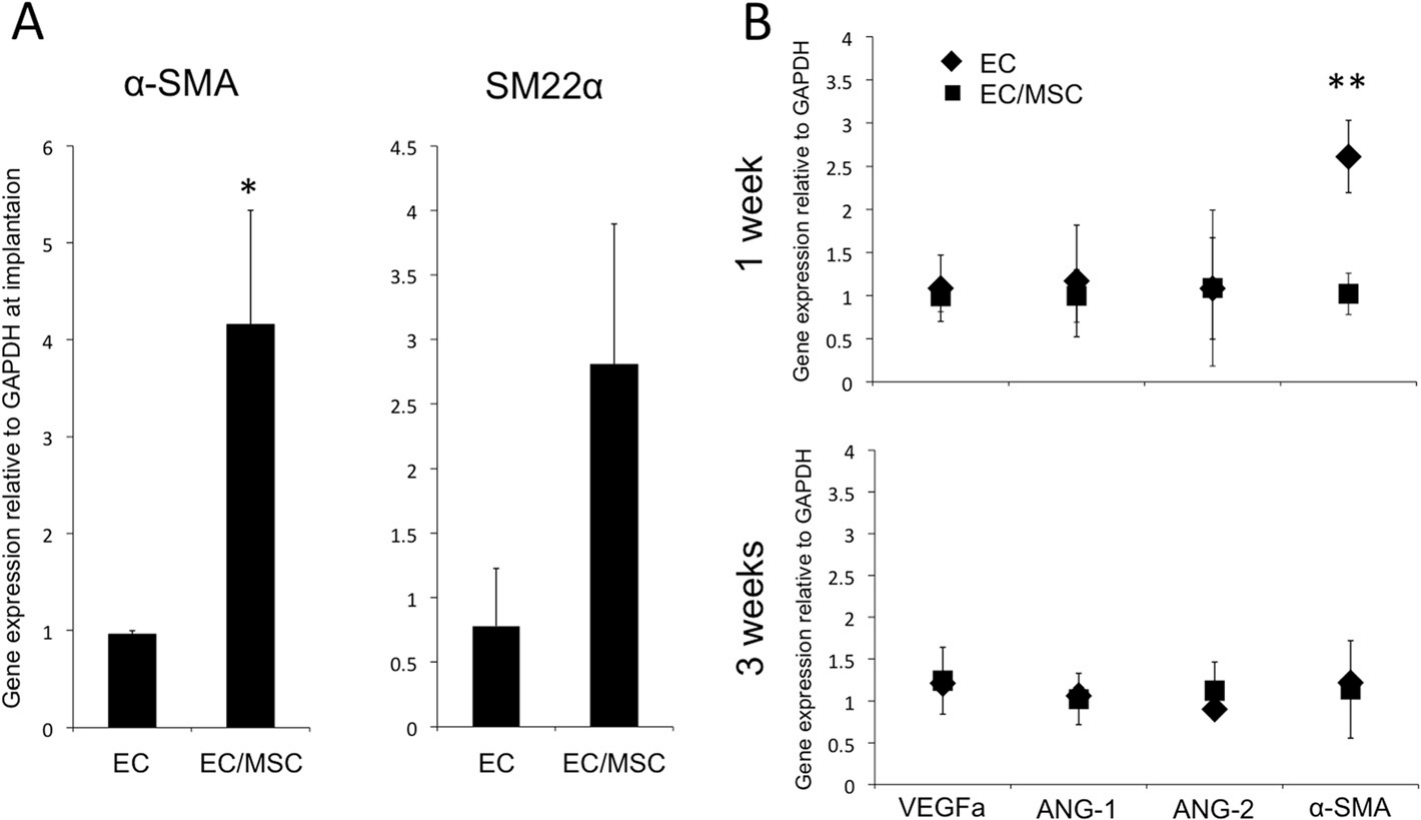
**Human and mouse specific perivascular gene expression before and after implantation. (A)** The expression of human specific perivascular biomarkers α-smooth muscle actin (α-SMA) and SM22α were both up-regulated in co-culture before implantation. **(B)** There were no differences in the expression of the angiogenic factors VEGFa, ANG-1 and ANG-2 using mouse specific assays at either time point *in vivo*. Expression of mouse α-SMA was up-regulated one week post-implantation in the EC-group, the opposite regulation as was observed with the human specific α-SMA assay. No differences were found for mouse α-SMA three weeks post-implantation. ** = *P* <0.01. ANG-1, angiopoietin-1; ANG-2, angiopoietin-2; EC, endothelial cell; VEGFa, vascular endothelial growth factor-a.

Perivascular gene expression was evaluated through expression of α-SMA and SM22α, and increased expression of both markers was detected for EC/MSC constructs (Figure 3A). Evaluation of mouse specific vascular genes showed an up-regulated expression of α-SMA in mono-cultured ECs one week after implantation, whereas expression levels of VEGFa, ANG-1 and ANG-2 were not significantly different between the experimental groups (Figure 3B, upper). Three weeks after implantation, no differences were observed for the vascular target genes evaluated (Figure 3B, lower).

### Expression of vascular endothelial growth factor from human cells

**Figure 4:**
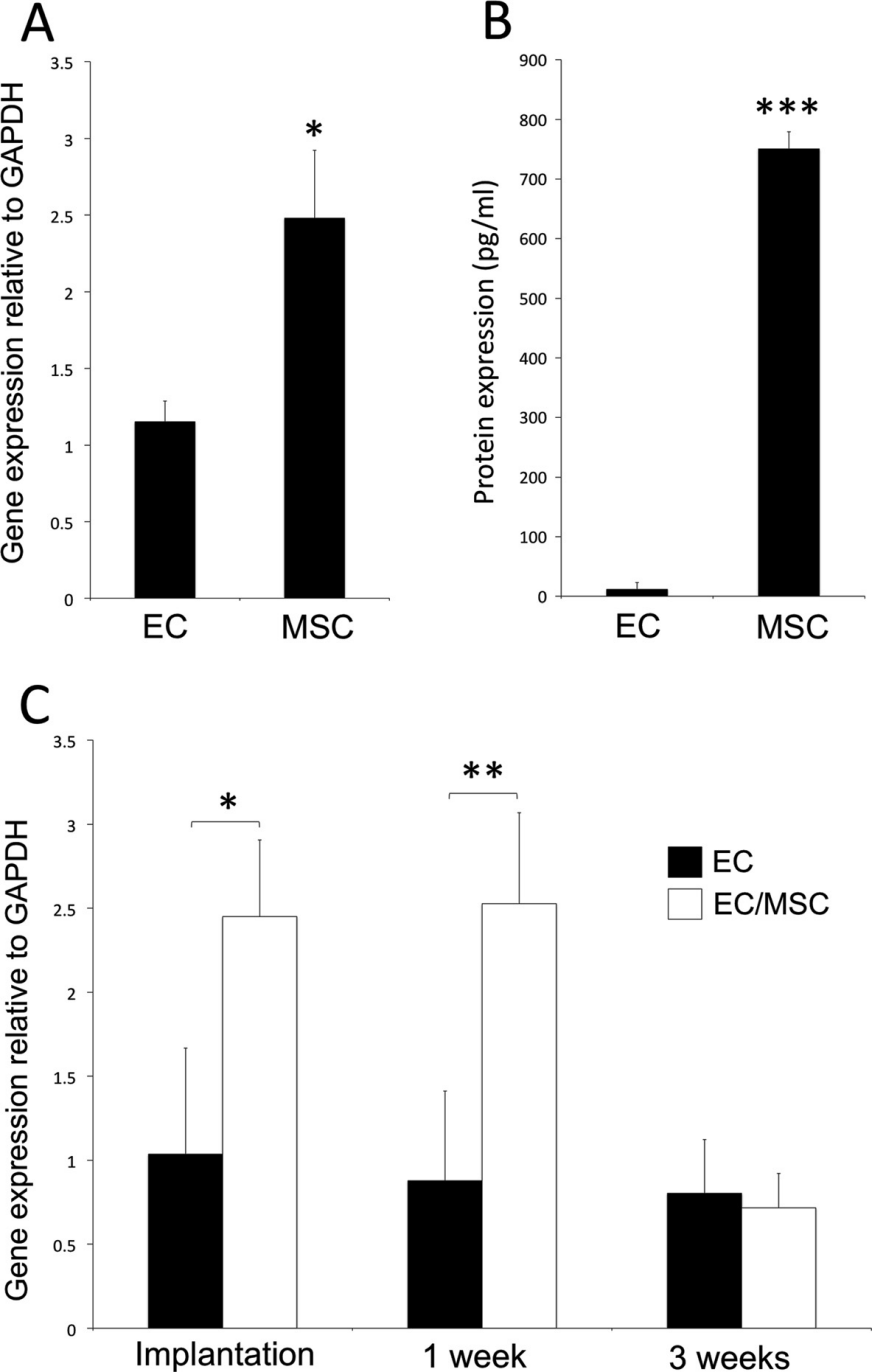
**Expression of vascular endothelial growth factor-a (VEGFa) from human cells.** Baseline gene **(A)** and protein **(B)** expression of VEGFa was significantly higher for MSCs compared with ECs grown in two-dimensional culture *in vitro*. **(C)** The relative gene expression of VEGFa from co-cultured constructs was also higher one week after dynamic three-dimensional culture *in vitro* and one week after implantation *in vivo*. * = *P* <0.05, ** = *P* <0.01, *** = *P* <0.001. ECs, endothelial cells; MSCs, mesenchymal stem cells.

Baseline mRNA (Figure 4A) and protein (Figure 4B) expression of VEGFa was evaluated for mono-cultured ECs and MSCs, and it was found that MSCs expressed significantly more VEGFa than ECs. Expression of VEGFa was evaluated at three time points: one week *in vitro* culture before implantation and one and three weeks after implantation *in vivo* (Figure 4C). For cell/scaffold constructs cultured *in vitro*, the expression of VEGFa was up-regulated in the co-culture system, and this was maintained one week post-implantation. Three weeks after implantation no significant differences were observed.

### Influence of implanted cells on inflammation and establishment of the mouse vasculature

**Figure 5:**
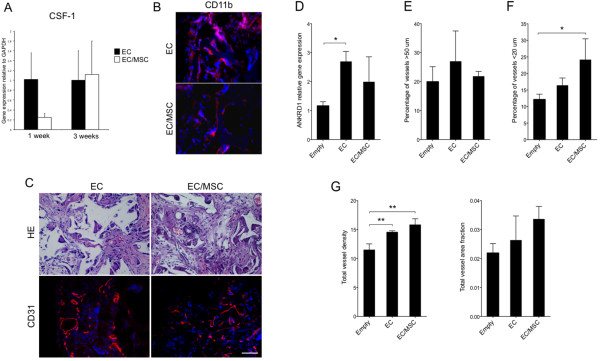
**Inflammation and vascularization of scaffolds after three weeks implantation. (A)** Early expression of human specific macrophage colony stimulating factor-1 (CSF-1) one week after implantation was higher in mono-culture, although not statistically significant (*P* = 0.079). Three weeks after implantation a difference could no longer be found. **(B)** Staining for CD11b, a protein subunit of the macrophage-1 antigen, was positive on the surface of the implanted constructs three weeks after implantation for both experimental groups. **(C)** Functional blood vessels were identified on H & E staining by the presence of erythrocytes in the lumen and through CD31-staining. Nuclei were stained with DAPI (blue). 20x magnification, scale bar = 100 μm. **(D)** The expression of ankyrin repeat domain 1 (ANKRD1) associated with EC activation was up-regulated in mono-cultured ECs compared with empty controls. * = *P* <0.05. **(E)** The distribution of vessels three weeks after implantation showed a higher percentage of vessels >50 μm in the EC-group: however, it was not statistically significant. **(F)** The percentage of vessels <20 μm was highest in EC/MSC-constructs. * = *P* <0.05. **(G)** Both cellular constructs had a higher vascular density at the end of the experimental period compared with empty control scaffolds. ** = *P* <0.01. The difference between the experimental groups was not statistically significant. The same tendency was observed for the total area fraction of CD31+ cells, but statistically significant differences were not found. DAPI, 4',6-diamidino-2-phenylindole; ECs, endothelial cells; MSC, meschymal stem cells.

The expression of human specific CSF-1 was higher in mono-culture one week post-implantation, although not statistically significant (*P* = 0.079). After three weeks this tendency could no longer be found (Figure 5A). CD11b + cells were found in close proximity to cell/scaffold constructs in both experimental groups three weeks after implantation (Figure 5B).

The expression of mouse specific ANKRD1 was used as a biomarker for activation of the mouse endothelium, and results show up-regulated expression in mono-culture compared to empty controls (Figure 5D). The difference between the experimental groups was not statistically significant. As regulation of vessel diameter is an important step in vascular maturation, blood vessels <20 μm were defined as mature microvessels, whereas vessels >50 μm were defined as immature. The remaining blood vessels were defined as transitional. A higher percentage of vessels <20 μm and >50 μm were found for both constructs where human cells were implanted (Figure 5E and F). The percentage of mature vessels (<20 μm) was highest in the EC/MSC-constructs, and the highest density of immature vessels (>50 μm) was detected in the EC group. Both cellular constructs had a higher density of blood vessels and area fraction of the total vasculature compared with the empty controls (Figure 5G). The vessel area fraction, however, was not statistically significant. Functional blood vessels were identified by the presence of erythrocytes in the lumen, and vascularization was evaluated through immunostaining for CD31 (Figure 5C).

## Discussion

In the process of vascular assembly, nascent vessels depend on interaction with perivascular cells to establish an endothelial-perivascular complex regulating vessel growth and maturation. Bioengineered tissues depend on mature blood vessels in order to adequately meet their metabolic demands, and perivascular cells are needed to generate a functional microvasculature. The present study shows how a quiescent phenotype of ECs was induced, by adding a low percentage of MSCs to the culture system. Biomarkers for vascular maturation and angiogenesis were both regulated by MSCs. In addition, MSCs were found to be a potent producer of VEGFa, an essential growth factor in vascular development. At the end of the experimental period, both cell/scaffold constructs induced a higher vascular density than controls, with the highest density of mature vessels being generated in constructs comprising ECs and MSCs.

Expression of endothelial specific ANG-1 and ANG-2 was altered in the presence of MSCs, with an up-regulation of ANG-1 and down-regulation of ANG-2. Competitive binding to the Tie2 receptor for both ligands is well known, and ANG-1 mediates vascular maturation by stabilizing ECs in the quiescent G_0_-phase, maintaining interactions between ECs, pericytes and the extracellular matrix [19,20]. ANG-2 is associated with destabilized ECs characteristic for angiogenic sprouting, thus having the opposite effect on the vascular endothelium. Proliferation and differentiation of cells are two processes that tend not to occur simultaneously. In order for nascent vessels to mature into patent tubes with controlled permeability, proliferation of ECs should subside [21]. On the gene level, the expression of cell proliferation biomarkers PCNA and ki67 was determined, with both genes being down-regulated after co-seeding with MSCs. In addition, cell counting revealed fewer viable cells in the presence of MSCs after one week of culture. Our results suggest the presence of MSCs to induce a quiescent phenotype of ECs, via decreased cell proliferation and angiopoietin-expression characteristic of vascular maturation. A potential interpretation error of results after three-dimensional co-culture was to isolate the expression patterns from the two cell types, as retrieval and separation of cells after culture in this highly interconnected scaffold has proven to be difficult. A combination of two- and three-dimensional culture could, therefore, provide more information, as has been performed in comparable experiments [22], in addition to analyzing factors differentially expressed for the two cell types, such as ANGs, CD31 and vWF (ECs), and pericyte markers (MSCs).

The recruitment of pericytes regulates the transition from vascular morphogenesis to a stabilized vasculature [7]. An increasing consensus regarding a perivascular origin for MSCs has emerged in stem cell biology [4], and the expression of perivascular markers has also been found for undifferentiated MSCs [23,24]. With up-regulated expression of both α-SMA and SM22α, one might suggest that ECs have an inductive effect on perivascular differentiation of MSCs. Certainly, biological factors involved in formation of an endothelial-perivascular complex were present in the EC/MSC-group before *in vivo* implantation. A comparison with mono-cultured MSCs might have been of further interest to strengthen this conclusion, although induced mural-cell differentiation of MSCs has been shown to be regulated by the gap junction Connexin-45, thus depending on direct contact between the two cell types [25]. Regardless of a terminal smooth muscle fate, MSCs have shown the ability to support development of endothelial microvascular networks in direct co-culture systems [15,24,26,27]. Enhanced proliferation of ECs has been reported following indirect co-culture [26,28], findings supported by the high expression of VEGFa detected from MSCs in the present work, suggesting the vascular assembly itself to be the event that induces the reduced endothelial cell proliferation, rather than secreted factors.

Regeneration in tissue engineering is likely to be caused by both differentiation of implanted cells and subsequent production of extracellular matrix proteins, as well as stimulation of surrounding tissues through the release of paracrine factors. Implantation of labeled cells could provide valuable information in animal models, but this strategy is difficult to apply in humans. The fate of MSCs labeled with luciferase has been followed through bioluminescent imaging subcutaneously and in a spine fusion model in rats [29]. Cells were shown to survive at least two weeks *in vivo*, but with a decreasing bioluminescent signal after one week. This has been supported in a similar subcutaneous model, as applied in the present work, where implanted MSCs did not survive more than three weeks after implantation [30]. Without perivascular cells, vascular maturation is impaired leading to an endothelium prone to regression [10,11]. However, signaling to surrounding tissues can still be substantial, and degenerating cells can deliver strong paracrine signals triggering a particular biological response.

When investigating the effect of cell implantation on the expression of angiogenic factors using mouse specific assays, an increase in α-SMA was found for mono-cultured ECs one week post-implantation. This was the opposite regulation as found for α-SMA expressed by implanted cells, which were higher with MSCs in the construct. The possibility for a compensatory down-regulation with implantation of perivascular MSCs could be suggested. The ANKRD1-gene is associated with EC activation and was used as a predictor of mouse specific endothelial activation. At the end of the three-week experimental period the expression was higher for mono-cultured ECs, which correlated with the largest percentage of immature vessels (>50 μm) also being detected in this group. Both findings support an increased vascular maturation achieved through co-implantation of ECs and MSCs. Endothelial cell density has been reported as a determining factor for mRNA expression of ANKRD1, with a high density of ECs associated with down-regulated ANKRD1-expression [31]. We also report here a higher area fraction of CD31+ cells in the EC-group and lower expression of ANKRD1. The other selected vascular factors evaluated from the animal tissue were unchanged, and the pro-angiogenic effects observed could therefore be attributed to the implanted cells, rather than the surrounding tissues. Alternatively, the inflammatory response generated by implantation of human cells contributed to increased expression of angiogenic factors and subsequent vascularization. Although NOD/SCID-mice are unable to undergo VDJ-recombination, we demonstrate the presence of CD11b + cells adjacent to cell/scaffold constructs. Induction of a CD11b + cell population preceding increased angiogenesis has previously been shown in a xenograft tumor transplantation model [32]. In the present work, recruitment of CD11b + cells was similar for both experimental groups, suggesting the xenograft model itself to be the primary causative agent, rather than a particular cell type. MSCs have been shown to possess immunomodulatory properties when transplanted *in vivo*[33], even suggesting MSCs as a therapeutic agent for autoimmune disease [34]. A minor down-regulation in the expression of CSF-1 was noted for EC/MSC-constructs one week after implantation, but a higher percentage of MSCs might have been needed to make a substantial effect on the immunological response. Overall, the expression of factors promoting angiogenesis was higher for mono-cultured ECs, such as pro-angiogenic and pericyte recruiting factors PDGFb and FGF-1. This could be due to the induced quiescence in the co-culture group with ECs in the resting G_0_-phase. However, perhaps the most important factor in vascular development, VEGFa, was significantly higher expressed in MSCs compared with ECs, and subsequently in EC/MSC-constructs. This is in accordance with previous work, where the supernatant from cultured MSCs induced sprouting of ECs [35]. At the end of the experimental period, the vascular density was higher in the co-culture group, which was not expected, due to the reduced activity of ECs. It could be suggested that the importance of VEGF, in fact, makes MSCs constitute a stronger trigger for vascular ingrowth than ECs, itself encouraging for tissue regeneration strategies with MSCs. The higher percentage of mature vessels <20 μm suggests that implantation of ECs with MSCs provides a favorable microenvironment for vascular maturation in a tissue-engineering context.

## Conclusions

In conclusion, the presence of MSCs even in low numbers induced a quiescent phenotype of ECs, regulating biomarkers for vascular maturation. Implantation of cell/scaffold constructs induced a higher vascular density than control scaffolds, whereas the highest density of capillaries was achieved through co-seeding of ECs and MSCs. Hence, MSCs can be appropriate perivascular cells for tissue-engineered constructs.

## Abbreviations

α-SMA: α-smooth muscle actin; ANG-1: angiopoietin-1; ANG-2: angiopoietin-2; ANKRD1: ankyrin repeat domain 1; CSF-1: macrophage colony stimulating factor-1; ECs: endothelial cells; ELISA: enzyme-linked immunosorbent assay; FGF-1: fibroblast growth factor-1; MSCs: mesenchymal stem cells; NOD/SCID-mice: non-obese severe combined immunodeficient mice; PCNA: proliferating cell nuclear antigen; PDGFb: platelet derived growth factor-b; PFA: paraformaldehyde; poly(LLA-co-DXO): poly(L-lactide-co-1,5-dioxepan-2-one); RT-PCR: reverse transcriptase-polymerase chain reaction; SMCs: smooth muscle cells; VEGFa: vascular endothelial growth factor-a; vWF: von Willebrand factor.

## Competing interests

The authors declare that they have no competing interests.

## Authors’ contributions

TOP, ALB, ZX, YX, YS, AFW, JBL, KNL, IF and KM conceived and designed experiments. TOP, ALB, ZX, YX and YS performed experiments. TOP, ALB, YX and KM analyzed data. AFW, JBL and KM contributed reagents, materials and analytical tools. TOP, ALB, KNL, IF and KM wrote the manuscript. All authors read and approved the final manuscript.
